# Elastic band resistance training influences transforming growth factor-ß receptor I mRNA expression in peripheral mononuclear cells of institutionalised older adults: the Vienna Active Ageing Study (VAAS)

**DOI:** 10.1186/s12979-016-0077-9

**Published:** 2016-06-30

**Authors:** Barbara Schober-Halper, Marlene Hofmann, Stefan Oesen, Bernhard Franzke, Thomas Wolf, Eva-Maria Strasser, Norbert Bachl, Michael Quittan, Karl-Heinz Wagner, Barbara Wessner

**Affiliations:** Research Platform Active Ageing, University of Vienna, Althanstraße 14, 1090 Vienna, Austria; Department of Sports and Exercise Physiology, Centre for Sport Science and University Sports, University of Vienna, Auf der Schmelz 6, 1150 Vienna, Austria; Karl Landsteiner Institute for Remobilization and Functional Health/Institute for Physical Medicine and Rehabilitation, Kaiser Franz Joseph Hospital, Social Medical Centre - South, Kundratstrasse 3, 1100 Vienna, Austria; Department of Nutritional Sciences, Faculty of Life Sciences, University of Vienna, Althanstraße 14, 1090 Vienna, Austria

**Keywords:** Vienna Active Ageing Study (VAAS), TGF-β pathway, microRNA, Chronic inflammation, Inflammageing, Strength training

## Abstract

**Background:**

Ageing, inactivity and obesity are associated with chronic low-grade inflammation contributing to a variety of lifestyle-related diseases. Transforming growth factor-β (TGF-β) is a multimodal protein with various cellular functions ranging from tissue remodelling to the regulation of inflammation and immune functions. While it is generally accepted that aerobic exercise exerts beneficial effects on several aspects of immune functions, even in older adults, the effect of resistance training remains unclear. The aim of this study was to investigate whether progressive resistance training (6 months) with or without nutritional supplementation (protein and vitamins) would influence circulating C-reactive protein and TGF-β levels as well as TGF-β signalling in peripheral mononuclear cells (PBMCs) of institutionalised adults with a median age of 84.5 (65.0–97.4) years.

**Results:**

Elastic band resistance training significantly improved performance as shown by the arm-lifting test (*p* = 0.007), chair stand test (*p* = 0.001) and 6-min walking test (*p* = 0.026). These results were paralleled by a reduction in TGF-β receptor I (TGF-βRI) mRNA expression in PBMCs (*p* = 0.006), while circulating inflammatory markers were unaffected. Protein and vitamin supplementation did not provoke any additional effects. Interestingly, muscular endurance of upper and lower body and aerobic performance at baseline were negatively associated with changes in circulating TGF-β at the early phase of the study. Furthermore, drop-outs of the study were characterised not only by lower physical performance but also higher TGF-β and TGF-βRI mRNA expression, and lower miRNA-21 expression.

**Conclusions:**

Progressive resistance training with elastic bands did not influence chronic low-grade inflammation but potentially affected TGF-β signalling in PBMCs through altered TGF-βRI mRNA expression. There appears to be an association between physical performance and TGF-β expression in PBMCs of older adults, in which the exact mechanisms need to be clarified.

## Background

Population ageing resulting from a higher life expectancy concomitant with a decline in fertility will lead to a global increase in the proportion of people over the age of 60 years from 12 % in 2015 to 22 % by 2050 [1]. With higher ages, the prevalence of cardiovascular, metabolic and neurodegenerative disorders and cancer is increased. It has been shown that many of these conditions are related to compromised homeostasis between proper activation of the immune system and its resolution, leading to a limited response to pathogens and vaccines and a chronic inflammatory state [2, 3].

Transforming growth factor-β (TGF-β) is a multifunctional protein involved in the regulation of cell proliferation, extracellular matrix production, inflammation and immune functions [4]. Higher circulating levels of TGF-β are related to obesity with impaired insulin sensitivity [5], cardiovascular diseases among individuals with higher C-reactive protein (CRP) levels [6], type II diabetes [7, 8], and a higher age [9]. TGF-β interacts with several cell types by binding to membrane serine/threonine kinase receptors, TGF-β receptor I (TGF-βRI; also known as ALK5) and TGF-βRII, initiating diverse cellular responses [10]. An approach to modulate TGF-β signalling is to alter the expression of its receptors. It has become evident that microRNAs (miRNAs) regulate thousands of human genes by either mRNA degradation or suppression of mRNA translation [11]. A recent investigation estimated that 84–89 % of miRNA-induced suppression of gene expression is the result of degradation of target mRNAs [12]. Currently, close to 1900 human miRNA precursors are listed in the miRBase registry, which give rise to 2588 mature miRNAs [13, 14]. One of these miRNAs, miRNA-21, plays a crucial role in a plethora of biological functions and diseases including the development of cancer, cardiovascular diseases and inflammation [15]. Interestingly, miRNA-21 targets both TGF-βRI and TGF-βRII [16], and overexpression of miRNA-21 is associated with elevated levels of proinflammatory cytokines in a dominant-negative TGF-βRII mouse model [17].

Regular physical activity offers protection against, and may be a useful treatment for, a wide variety of chronic diseases associated with low-grade inflammation [18]. In particular, endurance exercise training combined with dietary weight loss strategies have been shown to decrease the chronic inflammatory state associated with obesity and old age [19, 20]. Resistance training prevents or counteracts age-related loss of muscle mass and functions [21, 22], but there is an ongoing discussion whether this type of exercise alters the chronic inflammatory state associated with ageing [23–25].

In the Vienna Active Ageing Study (VAAS), we previously showed that resistance training using elastic bands for 6 months leads to an increase in functional performance of the lower and upper extremities and improves muscle quality in older people [22, 26]. Furthermore, we detected higher CRP levels and lower TGF-βRI and TGF-βRII expression in peripheral blood mononuclear cells (PBMCs) of older women compared with younger women [27]. Therefore, the aim of this sub-study of the VAAS was to investigate whether progressive resistance training alone or in combination with a nutritional supplement containing protein and vitamins influenced expression of TGF-β, its receptors, and/or miRNA-21 in older institutionalised people.

## Results

### Subject characteristics

**Fig. 1:**
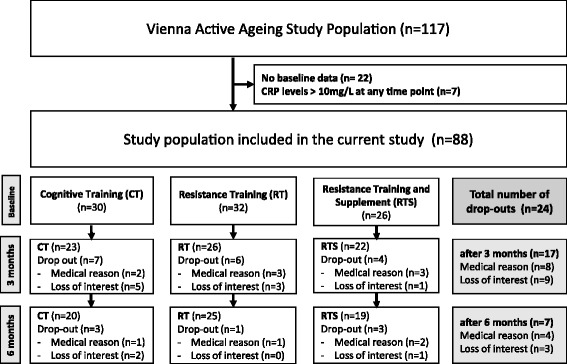
Participant flow

A total of 230 potential participants were screened for eligibility, and 117 participants (89 women and 12 men) agreed to participate in the VAAS. The participants were randomised into intervention groups: cognitive training (CT), resistance training (RT), and resistance training plus nutritional supplementation (RTS) (Fig. 1). Blood samples were available from 95 participants (84 women and 11 men). To prevent any potential interference by acute inflammatory processes, we excluded all subjects with CRP levels above 10 mg/L at any time point as done previously [28, 29]. Finally, data from 88 participants (77 women and 11 men) with a median age of 84.5 (65.2–97.4) years and a median body mass index (BMI) of 28.9 (18.1–50.0) kg/m^2^ were included in the current study (Fig. 1). No differences between study groups with respect to age, gender, anthropometric data and health status were detected at baseline, whereas leukocyte numbers differed significantly between intervention groups (*p* = 0.002). Post-hoc analyses revealed that subjects from the RT group had lower leukocyte numbers than those from the CT group (+28 %, *p* = 0.002) and the RTS group (+19 %, *p* = 0.039), which was owing to lower numbers of neutrophils (CT: +51 %, *p* < 0.001; RTS: +33 %, *p* = 0.092). Circulating inflammatory markers (hs-CRP and TGF-β) and mRNA expression of TGF-β, its receptors and miRNA-21 in PBMCs (lymphocytes and monocytes) did not differ between groups at baseline (Table 1).

**Table 1:** Baseline characteristics

	All (*n* = 88)	CT (*n* = 30)	RT (*n* = 32)	RTS (*n* = 26)	*P-*value
Gender [female/male]	77/11	25/5	28/4	24/2	0.602
Age [years]	84.5 (65.0–97.4)	84.5 (69.4–97.4)	84.4 (71.7–93.2)	84.3 (65.0–92.2)	0.864
BMI [kg/m^2^]	28.9 (18.1–50.0)	29.8 (18.1–36.9)	28.2 (22.7–40.2)	27.9 (22.9–50.0)	0.741
Obesity [n (%)]	32 (36.8 %)	14 (46.7 %)	8 (25.0 %)	10 (35.7 %)	0.235
Hyperlipidaemia [n (%)]	32 (36.4 %)	9 (30.0 %)	10 (31.3 %)	13 (46.4 %)	0.226
Diabetes Type II [n (%)]	14 (15.9 %)	6 (20 %)	7 (21.9 %)	1 (3.6 %)	0.132
Hypertension [n (%)]	70 (79.5 %)	26 (86.7 %)	27 (84.4 %)	17 (60.7 %)	0.100
Cardiac diseases [n (%)]	27 (30.7 %)	12 (40.0 %)	9 (28.1 %)	6 (21.4 %)	0.362
Osteoporosis [n (%)]	35 (39.8 %)	12 (40.0 %)	12 (37.5 %)	11 (39.3 %)	0.933
History of cancer [n (%)]	12 (13.6 %)	5 (16.7 %)	4 (12.5 %)	3 (10.7 %)	0.833
Leukocyte subpopulations and circulating biomarkers					
Leukocytes [x10^9^ cells/L]	6.6 (3.3–13.0)	7.4 (4.9–10.3)*	5.8 (3.1–9.8)	6.9 (4.4–13.3)*	**0.002**
Lymphocytes [cells/μl]	1990 (930–4930)	1985 (910–3510)	1835 (830–3330)	2200 (1460–4700)	0.085
Neutrophils [cells/μl]	3695 (1530–9860)	4414 (2410–6630)*	2931 (1530–6800)	3889 (2370–9860)	**<0.001**
Eosinophils [cells/μl]	200 (10–740)	225 (36–620)	160 (10–740)	220 (10–470)	0.135
Basophils [cells/μl]	40 (10–111)	45 (10–111)	35 (10–190)	35 (10–90)	0.397
Monocytes [cells/μl]	560 (250–910)	587 (72–950)	520 (260–860)	545 (320–960)	0.189
hs-CRP [mg/L]	1.95 (0.3–7.9)	2.1 (0.5–7.9)	1.9 (0.3–7.6)	1.9 (0.6–6.7)	0.918
TGF-β [μg/L]	33.3 (16.7–73.7)	38.2 (18.7–73.7)	32.8 (16.7–55.4)	32.1 (21.0–51.2)	0.083
PBMC gene expression					
TGF-β/GAPDH [-]	0.85 (0.06–3.36)	0.48 (0.23–2.66)	0.60 (0.21–2.52)	0.58 (0.06–3.36)	0.725
TGF-βRI/GAPDH [-]	2.05 (0.14–28.82)	1.99 (0.14–22.39)	2.04 (0.69–19.25)	2.11 (0.29–28.81)	0.990
TGF-βRII/GAPDH [-]	1.67 (0.51–15.85)	1.54 (0.66–7.79)	1.60 (0.66–14.85)	1.79 (0.51–10.80)	0.714
miRNA–21 [copies/pg]	2400 (57–4720)	2920 (343–4500)	2120 (57–4720)	2350 (550–4590)	0.765

Data are expressed as medians (min–max); Group differences were detected using *X* ^2^ or Kruskal–Wallis tests and if significant, followed by Bonferroni-corrected post-hoc analyses (*p < 0.05 vs. RT); significant differences are marked in bold

*Abbreviations*: *CT* cognitive training, *RT* resistance training, *RTS* resistance training plus supplement, *PBMC* peripheral blood mononuclear cell, *hs-CRP* high sensitive C-reactive protein, *TGF-β* transforming growth factor-β, *TGF-βR* TGF-β receptor, *GAPDH* glyceraldehyde 3-phosphate dehydrogenase, *miR-21* microRNA-21

### Dropout analysis

During 6 months of intervention, 18 women and 3 men (27 %) left the study because of several reasons (loss of interest or acute medical reasons such as joint pain or cardiological issues). The dropout rate did not differ between groups. In addition, age, sex, and existing co-morbidities in general were not related to adherence to the study. Only the number of participants diagnosed for hyperlipidaemia was higher in finishers compared with the dropouts (*p* = 0.016). Baseline performance appeared to be related to study adherence because dropouts had lower aerobic (6MWT: −19 %, *p* = 0.020) and chair stand (−13 %, *p* = 0.015) performance than finishers of the study. Strength (*p* = 0.608) and muscular endurance (*p* = 0.924) of the upper extremities were not different between finishers and dropouts. The observed differences also became evident in some of the TGF-β-related parameters because TGF-β (+106 %, *p* = 0.016) and TGF-βRI (+237 %, *p* < 0.001) mRNA expression was higher in dropouts, while miRNA-21 expression was lower (−65 %, *p* < 0.001) (Table 2). As a consequence we performed a per-protocol analysis by excluding the dropouts from all subsequent analyses (Tables 3 and 4).

**Table 2:** Baseline differences between drop-outs and finishers

	Total (*n* = 88)	Dropouts (*n* = 21)	Non-Dropouts (*n* = 67)	*p-*value
Gender [female/male]	77/11	18/3	59/8	0.777
Age [years]	84.5 (65.0–97.4)	85.2 (69.4–92.2)	84.2 (65.0–97.4)	0.282
BMI [kg/m^2^]	28.9 (18.1–50.0)	28.3 (18.1–43.7)	30.7 (23.4–50.0)	0.094
hs-CRP [mg/L]	1.9 (0.3–7.9)	2.0 (0.8–7.9)	1.9 (0.3–7.6)	0.413
TGF-β signalling				
TGF-β [μg/L]	33.3 (16.7–73.7)	39.3 (22.5–58.0)	32.8 (16.7–73.7)	0.178
TGF-β/GAPDH [-]	0.53 (0.06–3.36)	1.07 (0.21–3.36)	0.52 (0.06–2.66)	**0.016**
TGF-β RI/GAPDH [-]	2.05 (0.14–28.81)	6.41 (1.31–28.81)	1.90 (0.14–22.39)	**<0.001**
TGF-β RII/GAPDH [-]	1.67 (0.51–14.85)	3.00 (0.91–10.80)	1.59 (0.51–14.85)	0.099
miR–21 [copies/pg RNA]	2400 (57–5720)	979 (498–3640)	2815 (57–4720)	**<0.001**
Co-morbidities				
Obesity [n (%)]	32 (36.4 %)	10 (47.6 %)	22 (32.8 %)	0.162
Hyperlipidemia [n (%)]	32 (36.4 %)	3 (14.2 %)	29 (43.3 %)	**0.016**
Diabetes Type II [n (%)]	14 (15.9 %)	3 (14.3 %)	11 (16.4 %)	0.816
Hypertension [n (%)]	70 (79.5 %)	17 (81.0 %)	53 (79.1 %)	0.855
Cardiac diseases [n (%)]	27 (30.7 %)	7 (33.3 %)	20 (29.9 %)	0.763
Osteoporosis [n (%)]	35 (39.8 %)	9 (42.9 %)	26 (38.8 %)	0.741
History of cancer [n (%)]	12 (13.6 %)	3 (14.3 %)	9 (13.4 %)	0.921

Data for continuous variables are shown as medians (min–max). For co-morbidities, the respective numbers of affected participants were determined at the medical entrance examination. Obesity was defined as a BMI of >30 kg/m^2^. Differences between groups were determined by Mann–Whitney U or *X* ^2^ tests; significant differences are marked in bold

**Table 3:** Intervention effects on leukocyte subpopulations and circulating inflammatory markers

	Baseline (*n* = 67)	3 months (*n* = 67)	6 months (*n* = 67)	*P-*value (time)
Leukocytes [x10^9^ cells/L]				
CT (*n* = 26)	7.2 (4.9–10.3)^**^	7.2 (4.1–11.9)	7.1 (5.4–9.4)	0.440
RT (*n* = 20)	5.6 (3.1–9.1)	6.0 (3.2–8.9)	6.0 (3.3–9.8)^*^	**0.001**
RTS (*n* = 21)	7.0 (4.4–13.3)^**^	7.2 (4.5–11.7)^**^	7.1 (4.7–13.0)	0.586
Lymphocytes [cells/μl]				
CT (*n* = 26)	1985 (910–3510)	2090 (890–3980)	2250 (930–3540)	0.387
RT (*n* = 20)	1675 (830–3330)	1765 (912–3190)	1720 (1010–3430)	0.651
RTS (*n* = 21)	2255 (1460–4700)	2340 (1570–4680)	2190 (1640–4930)	0.810
Monocytes [cells/μl]				
CT (*n* = 26)	610 (72–950)	550 (300–780)	590 (400–910)	0.534
RT (*n* = 20)	520 (260–790)	475 (250–960)	510 (250–880)	0.104
RTS (*n* = 21)	585 (320–960)	490 (300–840)	545 (330–900)	0.404
Neutrophils [cells/μl]				
CT (*n* = 26)	4300 (2410–6630)^**^	4010 (1990–7420)	3910 (3110–5880)	0.247
RT (*n* = 20)	2876 (1530–6800)	3390 (1720–5880)	3520 (1800–7330)^*^	**0.011**
RTS (*n* = 21)	4030 (2430–9860)	3930 (1720–8730)	4290 (1664–8680)	0.368
Basophils [cells/μl]				
CT (*n* = 26)	40 (20–111)	30 (10–90)	30 (20–100)	**0.027**
RT (*n* = 20)	35 (10–90)	30 (10–100)	30 (10–80)	0.878
RTS (*n* = 21)	40 (10–90)	40 (10–90)	40 (10–80)	0.344
Eosinophils [cells/μl]				
CT (*n* = 26)	210 (36–448)	170 (80–470)	220 (90–330)	0.911
RT (*n* = 20)	160 (10–690)	186 (40–530)	200 (80–570)	0.381
RTS (*n* = 21)	248 (90–470)	195 (20–630)	260 (90–650)	0.137
hs-CRP [mg/L]				
CT (*n* = 26)	2.0 (0.5–4.8)	2.4 (0.2–5.5)	1.8 (0.3–8.5)	0.424
RT (*n* = 20)	1.9 (0.3–7.6)	2.4 (0.5–7.7)	2.0 (0.6–9.6)	0.645
RTS (*n* = 21)	1.8 (0.6–5.4)	2.1 (0.6–8.5)	1.6 (0.4–5.5)	0.135
TGF-β [μg/L]				
CT (*n* = 26)	36.0 (18.7–73.7)	39.8 (18.4–67.8)	40.8 (21.9–67.9)	0.953
RT (*n* = 20)	31.5 (16.7–50.0)	34.5 (18.3–59.7)	34.6 (12.7–57.3)	0.075
RTS (*n* = 21)	33.7 (21.0–51.2)	38.0 (22.9–50.5)	35.5 (22.1–52.1)	0.431

Data are expressed as medians (min–max); Differences over the time course were detected using the Friedman test and if significant, followed by Bonferroni-corrected post-hoc analyses (^*^ *p* < 0.05 vs. baseline within respective study group). Differences between groups were analysed by Kruskal Wallis test, followed by Bonferroni-corrected post-hoc analyses (^**^ *p* < 0.05 vs. RT group within respective time point; significant differences are marked in bold)

*Abbreviations*: *CT* cognitive training, *RT* resistance training, *RTS* resistance training plus supplement, *hs-CRP* high sensitive C-reactive protein, *TGF-β* transforming growth factor-β

**Table 4:** Intervention effects on TGF-β signalling gene expression

	Baseline (*n* = 67)	3 months (*n* = 67)	6 months (*n* = 67)	*P-*value (time)
TGF-β/GAPDH [-]				
CT (*n* = 21)	0.47 (0.23–2.66)	0.47 (0.27–4.10)	0.44 (0.25–1.93)	0.772
RT (*n* = 26)	0.54 (0.26–2.30)	0.52 (0.16–2.63)	0.52 (0.23–2.30)	0.568
RTS (*n* = 20)	0.55 (0.06–1.63)	0.45 (0.15–2.20)	0.56 (0.09–1.32)	0.692
TGF-βRI/GAPDH [-]				
CT (*n* = 21)	1.99 (0.14–22.39)	1.62 (0.46–29.89)	1.97 (0.11–9.24)	0.717
RT (*n* = 26)	1.87 (0.69–9.54)	1.61 (0.34–9.21)*	1.77 (0.54–15.91)	**0.006**
RTS (*n* = 20)	1.95 (0.30–8.63)	1.63 (0.28–8.59)	2.38 (0.52–6.73)	0.801
TGF-βRII/GAPDH [-]				
CT (*n* = 21)	1.54 (0.66–7.79)	1.39 (0.68–9.17)	1.48 (0.67–6.32)	0.827
RT (*n* = 26)	1.60 (0.66–14.86)	1.59 (0.57–13.50)	1.67 (0.63–8.79)	0.296
RTS (*n* = 20)	1.58 (0.51–5.13)	1.73 (0.44–3.98)	1.95 (0.69–4.73)	0.331
miRNA-21 [copies/pg RNA]				
CT (*n* = 21)	3163 (343–4500)	2840 (276–5120)	2460 (1050–4700)	0.861
RT (*n* = 26)	2602 (57–4720)	2340 (758–5600)	2540 (330–5020)	0.368
RTS (*n* = 20)	2536 (845–4590)	2535 (353–4780)	2810 (1600–4630)	0.854

Data are expressed as medians (min–max); Differences were detected using the Friedman test and if significant, followed by Bonferroni-corrected post-hoc analyses (* *p* < 0.05 vs. baseline; significant differences are marked in bold)

*Abbreviations*: *CT* cognitive training, *RT* resistance training, *RTS* resistance training plus supplement, *TGF-β* transforming growth factor-β, *TGF-βR *TGF-β receptor, *GAPDH* glyceraldehyde 3-phosphate dehydrogenase, *miR-21 *microRNA-21

### Resistance training improves physical performance of older subjects

We have previously shown that resistance training intervention specifically improved muscular endurance (chair stand and arm-lifting tests), while isometric strength (handgrip test) and aerobic capacity (6-min walking test; 6MWT) increased similarly in CT, RT and RTS groups [22]. Because the current study included just a subset of the original study population, the data on physical performance were re-analysed. At baseline, no differences in any of the physical fitness parameters were observed between intervention groups (*p* > 0.05). Similar to the previous results, chair stand performance was increased in the RT group (3 months: +18 %, *p* = 0.041; 6 months: +27 %, *p* = 0.001) and RTS group (6 months: +15 %, *p* = 0.017). Arm-lifting performance was enhanced in RT and RTS groups only after 6 months (RT: +24 %, *p* = 0.007; RTS +61 %, *p* = 0.007). While handgrip strength was unaltered over time in all intervention groups (CT: *p* = 0.454; RT: *p* = 0.238, RTS: *p* = 0.810), aerobic performance during the 6MWT was slightly improved in the RT group after 6 months (+9 %, *p* = 0.026) but not in the CT or RTS groups (CT: *p* = 0.959, RTS: *p* = 0.080) (data not shown).

### Effects of interventions on leukocyte subpopulations and circulating inflammatory markers

Except for an adaptation of the leukocyte numbers in the RT group (overall: *p* = 0.001; 3 months: *p* = 0.166; 6 months: *p* = 0.001; +7 %) caused by an increase in neutrophils (overall: *p* = 0.011; 3 months: *p* = 0.166; 6 months: *p* = 0.010 + 22 %), we did not detect any other alterations in leukocyte, lymphocyte, monocyte or granulocyte counts. Similarly, the circulating levels of TGF-β and hs-CRP remained unchanged (Table 3).

### Influence of interventions on intracellular TGF-β and TGF-β receptor mRNA expression, and miRNA-21 expression

The mRNA expression of TGF-β, TGF-βRI, TGF-βRII and miRNA-21 in PBMCs did not differ between groups at any time point. Resistance training led to a significant decrease in TGF-βRI mRNA levels (*p* = 0.006). Post-hoc analyses revealed that the initial decrease at 3 months (−27 %, *p* = 0.015) was reversed at 6 months (*p* = 0.117). Interestingly, this decrease could not be confirmed in the RTS group, although the median level decreased similarly to the RT group. Intracellular TGF-β and TGF-βRII mRNA expression, and miRNA-21 expression, were not influenced by any of the interventions (Table 4).

Because resistance training only led to marginal changes in the TGF-β signalling pathway, we examined whether different components of physical fitness (strength, muscular endurance and aerobic performance) at baseline influenced the TGF-β signalling response (difference between 3 or 6 months and baseline values). While isometric handgrip strength was not associated with alterations in TGF-β-related parameters, muscular endurance and aerobic performance at baseline were negatively associated with changes in circulating TGF-β levels after 3 months (chair rise test: *ρ* = −0.349, *p* = 0.003, arm-lifting test: *ρ* = −0.352, *p* = 0.024; 6MWT: *ρ* = −0.308, *p* = 0.009), but chair stand performance was positively associated with changes in TGF-βRII mRNA expression (*ρ* = 0.254, *p* = 0.033) (Table 5).

**Table 5:** Association between physical performance at baseline and the response of TGF-β-related parameters

		Baseline performance			
		Handgrip strength	Chair stand test	Arm lifting test	6-MWT
Change (difference between 3 months and baseline)	Circulating TGF-β	-0.039	-0.349*	-0.352*	-0.308*
	TGF-β/GAPDH	0.103	0.220	-0.051	0.199
	TGF-β RI/GAPDH	0.222	0.222	-0.148	0.148
	TGF-β RII/GAPDH	0.142	0.254*	-0.027	0.204
	miRNA-21	0.201	0.143	-0.029	0.038

Data indicate Spearman-Rho correlation coefficients. * *p* < 0.05 (significant correlations are marked in bold)

## Discussion

The aim of the current study was to investigate the effects of progressive resistance training alone and in combination with a nutritional supplement enriched with protein and vitamins on systemic inflammation and TGF-β signalling in PBMCs of institutionalised, but independent older people. Circulating TGF-β and hs-CRP levels as well as intracellular TGF-β gene expression were not influenced by elastic band resistance training. Interestingly, TGF-βRI, but not TGF-βRII, gene expression was reduced in the RT group after 3 months and returned to baseline levels thereafter.

It is well known that regular endurance training reduces the chronic proinflammatory state caused by ageing and physical inactivity [30, 31]. Adipose tissue appears to play a substantial role in this scenario because it is a major source of several hormones and cytokines [32]. In particular, visceral fat depots and the macrophages within these depots release proinflammatory cytokines such as interleukin-6 (IL-6), tumour necrosis factor-α (TNF-α) and TGF-β1 [33]. The increase in energy expenditure caused by enhanced physical activity reduces body fat, thereby influencing the capacity to produce and release proinflammatory mediators [33]. Recommended strategies to lose weight and body fat include aerobic exercise training combined with caloric reduction. Although it is known that resistance training does not promote clinically significant weight loss, it influences body composition by increasing muscle mass and decreasing body fat [34, 35]. Thus, it has been hypothesised that muscle-strengthening exercises also exert anti-inflammatory effects. Based on intervention studies investigating the effects of strength training on inflammatory markers such as CRP, TNF-α and IL-6, the data are ambiguous because some studies have revealed a positive effect [23, 36], whereas others did not observe any amelioration in the inflammatory state [24, 25, 37]. While the TGF-β superfamily has been studied extensively in the adaptation of muscles and tendons to exercise [38], investigations into the context of exercise immunology are scarce. Our data are in line with a previous study showing that strength training does not alter the level of circulating TGF-β [39]. Another study, investigating the influence of a combined strength and endurance exercise programme in type 2 diabetic patients revealed an increase in circulating TGF-β [36], but study population and training programme were different making a direct comparison difficult. Nevertheless, in our study, parameters measuring aerobic fitness and muscular endurance, but not handgrip strength, correlated negatively with TGF-β alterations. This could hint to the fact that endurance training is an important component of an exercise intervention to target circulating TGF-β levels, but further studies are needed to clearly identify the underlying associations.

While circulating levels of TGF-β were unaffected, our data revealed that resistance training seems to lower the TGF-βRI mRNA expression in PBMCs of older adults, potentially leading to decreased signalling through the type I receptor. Furthermore, higher levels of TGF-βR1 mRNA were detected in the less fit dropouts. As the mRNA was extracted from isolated PBMCs which include lymphocytes and monocytes, but not neutrophils or erythrocytes, it is very unlikely that the observed alterations in TGF-βRI mRNA are directly caused by varying neutrophil counts between groups. However, due to the pleiotropic effects of TGF-β, it is difficult to interpret the clinical implication of these findings. While TGF-β signalling is essential for regulatory T (T_reg_) cell maturation and immune homeostasis [40, 41], excessive signalling may lead to dysregulated T_Reg_ cell activity and may underlie a diverse range of allergic diseases in humans [42, 43]. Drugs which aim to block the TGF-β signalling are under investigation in connection with several disorders such as hypertrophic cardiomyopathy [44], hypertension [45] or the Marfan syndrome [46] and are suggested to be valuable for treating food allergies [42]. Therefore, the observed alterations in TGF-βRI mRNA expression caused by resistance training could be beneficial in these situations, whereby future studies need to clarify whether lower mRNA levels de facto lead to a lower expression of the receptor on the surface of the respective cells and whether these changes are clinically relevant.

It has to be mentioned that the median TGF-βR1 mRNA expression was slightly lower in both, the RT and the RTS group (-14 % and -16 %, respectively), but reached significance only in the RT group. For physical performance (chair stand and arm lifting performance) similar gains could be detected in both groups. A recent systematic review demonstrated that combining protein supplementation with resistance training is effective for eliciting gains in fat-free mass among older adults, but does not appear to further increase muscle mass or strength, which was similar to our study [47]. In addition to proteins, the supplement in this study contained several vitamins. Based on previous analyses, this supplement aids to increase the uptake of vitamin D and folic acid as well as the plasma levels of vitamin B_12_ and folic acid in erythrocytes [48, 49]. As some of the variables in this study (TGF-βRI mRNA expression, leukocyte numbers, 6MWT) were altered in the RT but not in the RTS group, it seems that the supplement prevented some of the responses to exercise. There is an ongoing discussion whether antioxidant supplementation may even blunt an exercise-induced training effect [50]. Evidence indicates that reactive oxygen species modulate TGF-β signalling. In turn, TGF-β increases the production of reactive oxygen species and suppresses antioxidant enzymes [51]. Therefore, it cannot be excluded that rigorous scavenging of free radicals impaired the TGF-β pathway response in the RTS group.

A reduction in TGF-βRI expression can be caused by either lower production or an increase in degradation of its mRNA. Post-transcriptional degradation of mRNAs often involves miRNAs specific for the respective target gene [12]. We have investigated miRNA-21 that has been shown to suppress TGF-βRI and TGF-βRII [16]. Additionally, circulating miRNA-21 levels are increased in men with a low aerobic capacity as measured by maximal oxygen uptake [52]. However, its levels were not enhanced in RT or RTS groups, suggesting that other mechanisms and/or other miRNAs are involved in the down-regulation of TGF-βRI in PBMCs by strength training [53].

One striking secondary outcome of this study is that TGF-β, TGF-βRI and potentially TGF-βRII mRNA expression at the beginning of the study were higher in drop-outs compared with finishers, while miRNA-21 expression was lower. Drop-outs were less physically fit than finishers. We confirmed that age, sex, body composition and the presence of several co-morbidities might not contribute to this effect, but the proportion of subjects with hyperlipidaemia was higher among finishers.

Although this study provides interesting data on TGF-β-related parameters in the context of inflammaging and exercise training, we also have to highlight some of its limitations. Because the study is a secondary analysis of a previously conducted trial [22], it is obvious that the data need to be confirmed in a future prospective study. Similar to other studies, we were interested in additive effects of the nutritional supplement to strength training rather than investigating the effects of the supplement alone. While this approach represents a best practice model in exercise nutrition, this study design has a limited explanatory power in describing the observed differences between the RT and RTS groups. Finally, the number of female participants outnumbered the male participants by a significant degree. It also has to be mentioned that the proportion of community-dwelling men at an age of 85 years in Vienna is 34 % [54], but the proportion of male individuals in Viennese retirement homes is 19 % for this age group [55] making our study population representative in terms of sex distribution of institutionalised older individuals in Austria. Although it was not possible to perform sub-group analyses because of the low numbers of men, their exclusion led to the same conclusion, indicating that the data are reliable at least for older women.

## Conclusions

Resistance training is beneficial, even in very old subjects, and potentially influences TGF-β signalling through altered receptor mRNA expression. Furthermore, adherence to the intervention was significantly related to alterations in the TGF-β pathway at baseline, but further information is needed to clearly understand the role of TGF-β in the context of inflammaging, resistance training and nutritional supplementation.

## Methods

### Participants and study design

The study was conducted using a randomised observer-blind design with three parallel groups, RT, RTS and CT. Briefly, participants were untrained, over 65 years of age with a Mini-Mental State Examination score of ≥23 and free of any medical conditions that would impair their participation in a resistance training study [56]. From a total of 117 participants in the VAAS, only those with available blood samples and a hs-CRP level below 10 mg/L at any time point were included in the current study (*n* = 88). A detailed description of the study design has been published previously [22].

### Interventions

Interventions were conducted for 6 months, and measurements were obtained at baseline, after 3 and 6 months. Twice a week, RT and RTS groups performed supervised progressive resistance training without using any equipment other than elastic bands and their own body weight. Each exercise session consisted of a general warm-up of 10 min, followed by a resistance training session (35–40 min) incorporating one to two exercises for each of the six main muscle groups (legs, back, abdomen, chest, shoulder and arms), and was completed by a cool-down routine. Following an adaptation phase of 4 weeks using low external resistance (yellow Thera-Band®, 1 set of 15 repetitions per exercise with a higher resistance only if the subject was obviously unchallenged) exercise intensity was progressively increased by changing the Thera-Band® from yellow to red and further to black. Additionally, the exercise volume was enhanced by increasing the number of sets from one to two. Rate of progression was based on individual improvements (band colour was changed if participant would have been able to perform two more repetitions in the second set. A detailed description has been provided previously [22]. Additionally to the exercises, participants from the RTS group were encouraged to drink a supplement that was distributed every morning as well as directly after the resistance training (in total nine times per week). One portion consisted of 20.7 g protein (55 En %; 19.7 g whey protein containing more than 10 g essential amino acids including 3 g leucine), 9.4 g carbohydrates (25 En %), 3.0 g fat (18 En %), 1.2 g fibre (2 En %), various vitamins such as 800 IU (20 μg) vitamin D, minerals and trace elements (FortiFit; Nutricia GmbH, Vienna, Austria). Total energy per drink was 150 kcal. Intake of the nutritional supplement was controlled at breakfast as well as after each training session. The CT group served as a control group and performed activities based on cognitive tasks (memory training) and coordinative tasks (such as manual dexterity) twice weekly to provide a timely effort which was equal to those of the RT and RTS groups, respectively [22, 57].

### Determination of body composition, physical fitness and health

Standing height was assessed using a commercial stadiometer (Seca, Hamburg, Germany), Body mass was evaluated with a digital scale (BWB 700; Tanita, Amsterdam, Netherlands) to the nearest 0.1 kg with subjects lightly dressed and barefoot. BMI was calculated by dividing the body mass in kilograms by height in meters squared. To measure strength of the upper extremities, participants performed two trials of an isometric handgrip strength test (kg) using a dynamometer (JAMAR compatible handgrip dynamometer adapted to handle various hand sizes) in a sitting position with an angle of 90° in the elbow [58]. Functional performance of the lower extremities was measured by a chair stand test, and that of the upper extremities by an arm-lifting test as described previously [22, 59]. Aerobic capacity was assessed by the 6MWT conducted on a 30-m shuttle track [60]. Global cognitive function was determined by the Mini-Mental State Examination [61]. Co-morbidities (hyperlipidaemia, hypertension, osteoporosis, cardiac diseases, history of cancer and diabetes mellitus) were determined at the medical entrance examination. Adiposity was defined by a BMI of >30 kg/m^2^ according to the World Health Organization criteria.

### Blood sampling and analyses

After overnight fasting, venous blood samples were obtained in a resting state between 06:30 and 08:00 using Z serum Clot Activator collection tubes (Vacuette®; Greiner Bio-One GmbH, Kremsmünster, Austria) to analyse circulating TGF-β and hs-CRP, EDTA tubes (Vacuette®; Greiner Bio-One GmbH) to determine leukocyte subpopulations and BD Vacutainer® CPT Tubes (Becton, Dickenson and Company, Schwechat, Austria) containing ~130 IU Na-Heparin and 2 mL Ficoll™ to isolate PBMCs.

Hs-CRP was quantified on a Cobas 8000 (Roche Diagnostics, Vienna, Austria). Leukocyte subpopulations were determined by flow cytometric analyses on a XE-2100 automated hematology system (Sysmex Austria GmbH, Vienna, Austria). TGF-β was analysed using a commercially available DuoSet development kit to perform an enzyme-linked immunosorbent assay (DY240; R&D Systems, Abingdon, UK) following the manufacturer’s instructions including plasma activation by acidification (pH 2) by adding hydrochloric acid (final concentration: 0.1 mmol/L) and neutralisation with sodium hydroxide (final concentration: 0.12 mmol/L) before measurement.

PBMCs were separated from red blood cells and neutrophils using BD Vacutainer® CPT Tubes (Becton Dickinson Austria, Vienna, Austria). The obtained pellet was carefully resuspended in 700 μL QIAzol Lysis Reagent (Qiagen, Hilden, Germany) and stored at −80 °C until analysis.

Total RNA including small RNAs was isolated using a miRNeasy Mini Kit (Qiagen, Hilden, Germany) following the supplier’s protocol. To prepare a miRNA-enriched fraction separated from the larger RNAs (>200 nt), we used an RNeasy MinElute Cleanup Kit (Qiagen). Reverse transcription of the miRNA-enriched fraction was performed using a miScript II RT Kit (Qiagen), whereas larger RNAs were reverse transcribed using a QuantiTect Reverse Transcription Kit (Qiagen).

TGF-β, TGF-βRI and TGF-βRII mRNA levels were determined using the respective primer pairs [Hs_TGFB1_1 (QT00000728), Hs_TGFBR1_1 (QT00083412) and Hs_TGFBR2_1 (QT00014350); Qiagen] in conjunction with a QuantiTect SYBR Green PCR kit (Qiagen). In addition, glyceraldehyde-3-phosphate dehydrogenase [Hs_GAPDH_2 (QT01192646)] served as the endogenous control to normalise the data. Quantification was performed on an Applied Biosystems® 7500 Real-Time PCR System.

MiRNA-21 expression levels were detected using a miScript Primer Assay specific for miRNA-21 [hs_miR-21_2 (MS00009079); Qiagen]. Quantification was performed on the Applied Biosystems® 7500 Real-Time PCR System.

### Statistical analyses

Data acquisition and processing were performed using commercial software (IBM SPSS 20). The Shapiro–Wilk test was used to test for a normal distribution. Because most of the variables were not distributed normally, the non-parametric Mann-Whitney *U*-test or the Kruskal-Wallis test were used to compare two or more independent groups, whereas the Friedman test was applied to detect changes over time in different intervention groups. To avoid bias by multiple testing, the results were Bonferroni-corrected for post-hoc analyses. Correlations between variables were identified by Spearman’s rank correlation coefficient. Data are shown as the median (minimum–maximum). Statistical significance was set at *p* < 0.05.

## Abbreviations

BMI, body mass index; CT, cognitive training; GAPDH, glyceraldehyde 3-phosphate dehydrogenase; hs-CRP, high sensitive C-reactive protein; IL-6, interleukin-6; miRNA, microRNAv; PBMCs, peripheral mononuclear cells; RT, resistance training; RTS, resistance training plus nutritional supplementation; TGF-β, transforming growth factor-β; TGF-βR, TGF-β receptor; TNF-α, tumour necrosis factor-α; T_reg_, regulatory T cells; VAAS, Vienna Active Ageing Study
